# Synthesis of ZnO Nanoflower Arrays on a Protrusion Sapphire Substrate and Application of Al-Decorated ZnO Nanoflower Matrix in Gas Sensors

**DOI:** 10.3390/s23125629

**Published:** 2023-06-16

**Authors:** Xin Zhao, Jang-Cheng Jheng, Ni-Ni Chou, Fang-Hsing Wang, Cheng-Fu Yang

**Affiliations:** 1 School of Information Engineering, Shanghai Zhongqiao Vocational and Technical University, Shanghai 201514, China; zhaoxin@shzq.edu.cn; 2 Graduate Institute of Optoelectronic Engineering, National Chung Hsing University, Taichung 402, Taiwan; rock302520@gmail.com; 3 Department of Chemical and Materials Engineering, National University of Kaohsiung, Kaohsiung 811, Taiwan; a1091402@mail.nuk.edu.tw; 4 Department of Aeronautical Engineering, Chaoyang University of Technology, Taichung 413, Taiwan

**Keywords:** ZnO nanoflower matrix, protrusion sapphire substrate, Al-decorated, gas sensors

## Abstract

In this study, we utilized a sapphire substrate with a matrix protrusion structure as a template. We employed a ZnO gel as a precursor and deposited it onto the substrate using the spin coating method. After undergoing six cycles of deposition and baking, a ZnO seed layer with a thickness of 170 nm was formed. Subsequently, we used a hydrothermal method to grow ZnO nanorods (NRs) on the aforementioned ZnO seed layer for different durations. ZnO NRs exhibited a uniform outward growth rate in various directions, resulting in a hexagonal and floral morphology when observed from above. This morphology was particularly evident in ZnO NRs synthesized for 30 and 45 min. Due to the protrusion structure of ZnO seed layer, the resulting ZnO nanorods (NRs) displayed a floral and matrix morphology on the protrusion ZnO seed layer. To further enhance their properties, we utilized Al nanomaterial to decorate the ZnO nanoflower matrix (NFM) using a deposition method. Subsequently, we fabricated devices using both undecorated and Al-decorated ZnO NFMs and deposited an upper electrode using an interdigital mask. We then compared the gas-sensing performance of these two types of sensors towards CO and H_2_ gases. The research findings indicate that sensors based on Al-decorated ZnO NFM exhibit superior gas-sensing properties compared to undecorated ZnO NFM for both CO and H_2_ gases. These Al-decorated sensors demonstrate faster response times and higher response rates during the sensing processes.

## 1. Introduction

Metal oxides have gained significant attention in recent years as promising gas sensor materials. This is primarily due to their advantageous characteristics, including low cost, convenient processing, and high sensitivity [1]. Metal oxide semiconductor gas sensors offer a range of benefits such as high sensitivity, significant signal output changes even in response to low concentration gases, long lifespan, rapid response rate, and excellent reversibility. These gas-sensing devices operate based on the principle of gas molecules interacting with the metal oxide, resulting in conductivity changes. Their unique advantages, such as high sensitivity, cost-effectiveness, ease of manufacturing, and compatibility with silicon microfabrication, have made them the focus of extensive research [1,2,3]. Although zinc oxide (ZnO) thin films are widely employed in gas sensors, their effectiveness is constrained by their limited surface-to-volume ratios. Moreover, these sensors usually demand high operating temperatures exceeding 300 °C. Nevertheless, ZnO remains one of the most extensively researched gas sensing oxides due to its capability to detect a range of combustible and toxic gases. Notably, it has been studied extensively for the detection of gases such as NO_2_ [4,5], C_2_H_5_OH [5], CO [6], and H_2_ [7]. Hydrogen gas sensors have gained considerable attention across various applications due to their remarkable attributes, including ultra-low power consumption, safe operation in flammable and explosive environments, and long lifespan. Among the various sensing materials, ZnO nanorods (NRs) have emerged as a promising candidate, possessing exceptional properties such as high sensitivity to surface-adsorbed oxygen, excellent electrical conductivity, high photosensitivity, light waveguiding capabilities, and a large surface-to-volume ratio. The development of high-performance gas sensors has become a focal point for researchers in the field of solid-state gas sensing in recent years. Gas sensors play a vital role in environmental monitoring, home safety, and chemical control.

Compared to bulk or thin film materials commonly used for gas sensing, nanorods or nanowires offer several advantages, including higher gas sensitivity and the ability to operate at lower temperatures. This is primarily due to their larger sensing area [8]. Therefore, this study aims to explore a simpler method for preparing a ZnO nanoflower matrix (NFM). One-dimensional ZnO nanostructures, characterized by their high surface-to-volume ratios, are considered a promising material for enhancing gas sensing performance [9]. Carbon monoxide (CO) is an inorganic compound gas that is both colorless and odorless. Despite its lack of noticeable characteristics, it is tasteless and highly toxic. Carbon monoxide (CO) has long been a significant safety concern in residential settings, as its presence in unburned gas or the incomplete combustion of gas appliances can lead to gas poisoning. Inhalation of CO can result in poisoning, as the gas can spread through the air. On the other hand, hydrogen (H_2_) is widely used in industrial applications and is highly combustible. Concentrations of H_2_ exceeding 4% in the atmosphere pose an explosion hazard. Consequently, the development of high-quality sensors with excellent sensitivity, response rate, and reversibility for both CO and H_2_ has become a focal point of research in recent years. In light of this, CO and H_2_ were chosen as the target gases for this study.

In a previous study by Wang et al., a hydrothermal method was employed to grow nanorods (NRs) and nanotubes, leading to the successful fabrication of gas sensors with excellent sensitivity and reversibility for detecting CO [10]. When assessing the sensor’s quality, two crucial factors are the sensitivity and response time, which are measured by the electrical resistance response value (response). Additionally, reversibility plays a vital role. A sensor is considered to have good reversibility if its electrical properties consistently exhibit significant differences and changes when exposed to specific gases and air. Moreover, the sensitivity and response time should yield stable and repeatable measurements alternately, indicating a reliable and reversible response. Wang et al. conducted a study where they synthesized ZnO NRs using a hydrothermal method and developed gas sensors capable of detecting gases such as H_2_, NH_3_, and CO. Their sensors exhibited good reversibility [11]. Additionally, research by Liao et al. indicates that ZnO NRs with smaller diameters demonstrate higher sensitivity due to their relatively larger surface area. This increased surface area enhances gas adsorption and reaction, resulting in better electrical response [12]. In our previous work, we utilized a patterned sapphire substrate with a matrix cavity as a template. An aluminum (Al) layer was used as a sacrifice layer, and a ZnO gel layer was applied as a seed layer using the spin coating method. The seed layer with an array-patterned protrusion structure was prepared and then transferred onto the Si substrate using the lift-off method, with the aid of an optical glue as a carrier [13]. While this method proved successful in synthesizing ZnO nanoflower arrays, it was found to be complex. Hence, the objective of this study is to explore a simplified approach for preparing ZnO nanoflower arrays. The major innovation of this research lies in the development of an array of ZnO nanostructures on a protrusion sapphire substrate. The technology employed in this investigation enables the synthesis of ZnO nanorods (NRs) in the form of a ZnO nanoflower matrix (NFM), thereby enhancing the contact area and improving the sensitivity of CO and H_2_ gas sensors.

To enhance the sensing capabilities of ZnO nanostructures, various metal-based nanoparticles have been employed as surface decorations. A study conducted by Alev et al. involved the decoration of ZnO nanorods (NRs) with copper (Cu) metal. They demonstrated the significant impact of Cu decoration on ZnO NRs when deposited on quartz crystal microbalance oscillators, resulting in high-performance sensing of H_2_S and HCN gases [14]. Another study by Mohammad and Drmosh focused on the decoration of ZnO nanostructures with platinum (Pt) and gold (Au). The decorated ZnO nanostructures exhibited improved H_2_ sensing performance compared to undecorated ZnO nanostructures [15]. Lalmuanchhana et al. conducted a study where they decorated ZnO monolayer materials with transition metals such as Fe, Co, Ni, and Cu. They compared the adsorption of CO and NO gas molecules on these decorated devices [16]. In a separate study, Dilova et al. utilized Pd as a decoration material for ZnO nanostructures. They observed that Pd-decorated ZnO nanostructures demonstrated enhanced sensitivity and feasibility for NH_3_ and CO gas sensing compared to undecorated ZnO nanostructures [17]. In our study, we aimed to enhance the sensing properties of the ZnO nanoflower matrix (NFM) by decorating it with aluminum (Al) nanoparticles. We compared the efficiencies of CO and H_2_ gas sensing between the Al-decorated and undecorated ZnO nanostructures and discovered that the Al-decorated ZnO NFM exhibited higher sensitivity to these gases compared to the undecorated ones.

## 2. Experimental Procedures

### 2.1. Prepare Substrate and Deposit ZnO Seed Layer

**Figure 1:**
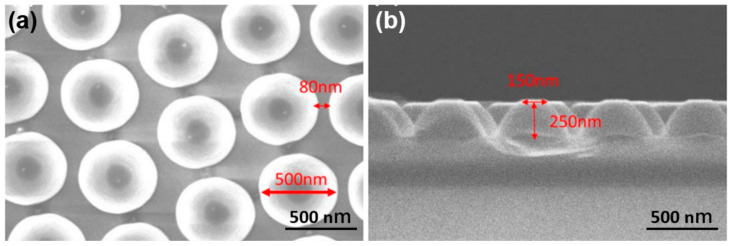
(**a**) Surface observation and (**b**) cross-sectional observation of protrusion sapphire substrate.

**Figure 2:**
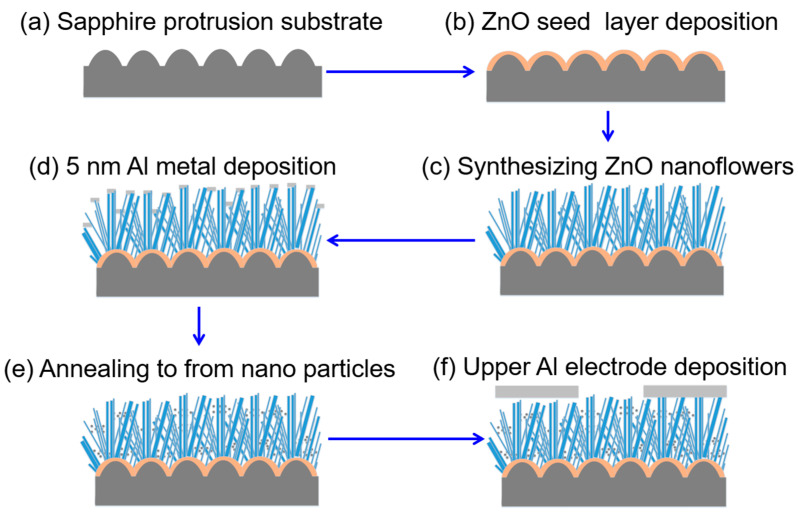
Schematic diagram for the fabrication processes of an Al-decorated ZnO nanoroads gas sensor.

In this study, we employed the hydrothermal method to synthesize ZnO nanorods (NRs) on a ZnO seed layer, resulting in the formation of a ZnO nanoflower matrix (NFM). The ZnO seed layer was prepared on a sapphire substrate, which served as a template with a protrusion matrix structure provided by Shun Haw Technology Ltd. (Changhua County, Taiwan). Figure 1a illustrates the top view of the 2-inch sapphire substrate, while Figure 1b shows its cross-sectional morphology. The concave nanostructures within the sapphire substrate had an average bottom width of 500 nm and a height of 250 nm. Figure 2a presents a schematic diagram illustrating the side view of the 2-inch sapphire substrate. The seed layers were coated using a spin coating method, following these steps:

(1) The sapphire substrate was thoroughly cleaned and positioned on the platform of the spin coating machine. A 10 mm × 10 mm area was selected for preparing the ZnO seed layer.
(2) The spin coating parameters were set as follows: a spin coating time of 30 s and a speed of 2000 rpm. These settings were specifically applied to the designated area on the substrate.
(3) A test spin was conducted to verify the speed and ensure proper attachment of the substrate to the machine.
(4) An appropriate quantity of ZnO gel solution was loaded into a syringe, which was then used to dispense drops of the solution onto the center of the sapphire substrate. The solution was uniformly spread over the entire surface of the substrate before initiating the spinning process.
(5) After the spinning step, the substrate was carefully removed and transferred to a baking plate. It was then subjected to a baking process at 300 °C for 10 min. This step aimed to dry and solidify the film while removing any residual organic solvent. Figure 2b illustrates this procedure. The aforementioned steps were repeated six times, with each repetition lasting 10 min, resulting in the formation of a ZnO seed layer consisting of six repeating layers.

### 2.2. Synthesize ZnO Nanorods, the Analyses of Their Crystallization, the Observations of Outward Appearances and Cross-Sectional Morphologies, and PL Analyses

In this experiment, ZnO NRs were synthesized using the hydrothermal method. Initially, ZnO seed layer, which had been grown on the sapphire substrate, was transferred onto a glass slide. The substrate, along with ZnO seed layer, was immersed in a Zn(CH_3_COO)_2_ solution, with the addition of C_6_H_12_N_4_ as a conditioning agent, both at the same concentration. Subsequently, the hydrothermal pot was heated to 90 °C and maintained at this temperature for 1 h. This allowed for the growth of ZnO NRs, with the growth time ranging from 30 to 60 min, as depicted in Figure 2c. Once the growth process was complete, the samples were carefully removed from the hydrothermal pot and rinsed with deionized water. This step aimed to eliminate any residual non-growth substances that might have been present on both the substrate and ZnO NRs.

Finally, the cleaned samples underwent a drying process at 90 °C to remove any residual water stains, thus completing the growth of ZnO NRs. The synthesized ZnO NRs on the patterned ZnO seed layer were subjected to analysis to determine their crystalline phases, using X-ray diffraction spectra. The surface morphologies of ZnO NR arrays were observed using field emission scanning electron microscopy (FESEM, Hitachi, Tokyo, Japan). High-resolution transmission electron microscopy (TEM, Jeol, Kyoto, Japan) was also used to find the crystallization of ZnO NRs. Additionally, we aimed to demonstrate that the growth of ZnO NRs on the patterned protrusion sapphire substrate resulted in the formation of a ZnO NFM. To examine the cross-sectional morphologies of both patterned ZnO seed layer and ZnO NFM, a focused ion beam system (FIB) was utilized. Furthermore, the Brunauer-Emmett-Teller (BET, Microtrac, York, PA, USA) analysis was employed as an effective technique to determine the specific surface area of the ZnO NFM. Additionally, the photoluminescence (PL) properties of ZnO NFM were evaluated using an iHR550 fluorescence spectrophotometer (Horiba, Kyoto, Japan). The measurements were conducted at room temperature with a single laser emitting at a wavelength of 325 nm. The PL spectra were recorded within the wavelength range of 350 to 650 nm. In order to analyze the chemical bonding state of oxygen, X-ray photoelectron spectroscopy (XPS) spectra were obtained. XPS is a valuable method for investigating the chemical bonding state in materials. In this study, XPS analysis was used to examine the effect of different synthesis times on the chemical bonding state of oxygen in ZnO NFM.

### 2.3. Prepare Al-Decorated ZnO Nanoflower Matrix and Gas Sensing

The deposition of the Al metal was carried out using the thermal evaporation method. The deposited Al film had a thickness of 5 nm, which was controlled by adjusting the deposition time and confirmed using a gauge measurement. Subsequently, the Al-deposited ZnO NFM underwent annealing at 550 °C for 10 min, resulting in the transformation of the deposited Al film into nanoparticles. This is illustrated in Figure 2d,e. To create the upper electrode, an Al metal layer with an interdigital structure was deposited onto ZnO NFM using the thermal evaporation method. Figure 2f provides a visual representation of this step. Before conducting measurements, the devices were subjected to a pretreatment process. Air was passed through the measurement chamber, and the temperature was raised to 350 °C for a duration of one h. 

To investigate the resistance changes of ZnO NFM sensing devices during gas measurements, a Keithley 2400 Source Meter was connected to a computer. The dynamic voltage signal generated by the Source Meter was applied to ZnO NFM sensing devices through the interdigital electrode. The computer controlled the output electrode signals, allowing for the measurement of resistance changes. The dynamic analyses of the resistance were monitored and recorded on the same computer. During the measurement process, different testing gases were introduced to observe their effects on the resistance of ZnO NFM sensing devices. The voltage applied to all the samples ranged from −5 V to +5 V, ensuring proper electrical contact between ZnO NFM and the Al electrodes. The I–V plots of the measurements demonstrated excellent ohmic behavior, indicating minimal junction resistance. Unfortunately, these plots are not included in the current discussion. The measurement chamber was evacuated to a pressure of 10^−2^ Torr, and the internal stage was heated directly to a temperature of 200 °C. The desired gas concentrations for the measurements were set and controlled by adjusting the inlet gas flow. The specific gas concentrations used in the experiment are listed in Table 1. Determining the gas concentration during the measurements involved considering factors such as the internal volume of the chamber, the concentration of gas in the cylinder, the flow rate of the mass flow control, and the ventilation time. The following formula was utilized for this calculation:

(1) $$\mathrm{Gc}\;(\mathrm{ppm})=\frac{MFC\times L\times 60}{S\times C(\%)\times 1000}$$

where Gc is the required gas concentration, where MFC represents the flow rate of the mass flow control in standard cubic centimeters per minute (sccm), L denotes the cavity volume, S represents the ventilation time, and C is the fixed value of the gas cylinder concentration. By adjusting the flow rate of the mass flow control and the feeding time, the concentration of the sensing gas to be measured can be set accordingly. The specific gas concentrations used in the experiment are presented in Table 1. The resistance response rate is defined as follows:

(2) $$\mathrm{Response}\;\mathrm{rate}=\frac{resistance\;in\;air\;(Ra)}{Resistance\;in\;sensing\;gas\;(Rg)}$$

## 3. Image Processing Results and Discussion

**Figure 3:**
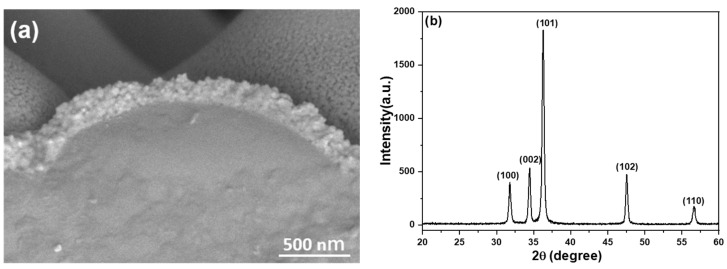
(**a**) SEM images and (**b**) XRD pattern of ZnO film deposited on the protrusion sapphire substrate.

A ZnO seed layer was prepared by performing spin coating on the protrusion sapphire substrate, with a total of six coating cycles. Subsequently, the spin-coated ZnO gel underwent annealing at 300 °C, resulting in the formation of nano-crystalline particles, as illustrated in Figure 3a. The resulting film had an average thickness of approximately 170 nm, with a deposition rate of around 28.3 nm per coating cycle. This information indicates the growth rate of ZnO seed layer during the spin-coating process. The X-ray diffraction (XRD) pattern of ZnO seed layer is presented in Figure 3b. The pattern reveals a prominent diffraction peak corresponding to the (101) plane, rather than the (002) plane. Additional diffraction peaks are observed on the (100), (102), and (110) planes. These findings indicate that ZnO seed layer does not exhibit a preferred orientation of the c-axis. The size of crystalline particles in the nanoscale can be determined using the Debye–Scherrer equation, which is represented by D = (k λ/β cosθ). In this equation, D represents the size of the crystalline particles, β is Scherer’s constant (0.94), λ is the X-ray diffraction wavelength (1.54178 Å), and θ is the angle corresponding to the (002) diffraction peak observed in Figure 3b. By inputting the FWHM (full width at half maximum) and θ values of the (002) diffraction peak into the Debye–Scherrer equation, the grain size of ZnO seed layer can be directly calculated. Based on the calculations, the grain size of ZnO seed layer is estimated to be approximately 54.9 nm. 

**Figure 4:**
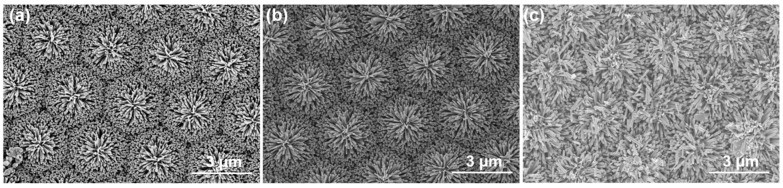
SEM top view of synthesized ZnO nanoroads, (**a**) 30, (**b**) 45, and (**c**) 60 min.

Figure 4 depicts the top view of synthesized ZnO NRs as observed through scanning electron microscopy (SEM). From the visual representation provided in Figure 4a,b, it is evident that ZnO NRs do not exhibit upward growth. Instead, they grow outwardly. This observation indicates that the growth directions of ZnO NRs are perpendicular to the ZnO seed layer, which is formed by the ZnO gel coated on the protruding sapphire substrate. Figure 4a,b present a significant finding: the outward growth rates of ZnO NRs in different directions are nearly identical. Consequently, when viewed from the top, ZnO NRs exhibit a hexagonal appearance, highlighting the uniformity of their growth. While the hexagonal prism structure of ZnO NRs is evident, the precise reason for the distinctive appearance of an equilateral hexagon is not fully understood. However, one possible explanation is that as ZnO NRs grow, they undergo competition in all directions, leading to the initial formation of a hexagonal shape with a balanced distribution of energy. Furthermore, the grown ZnO NRs exhibit a hemisphere-like shape with outwardly protruding needle-like structures, ultimately giving rise to the formation of ZnO nanoflowers. 

The specific mechanism behind this transformation and the factors influencing the shape and growth pattern of ZnO NRs and nanoflowers require further investigation. The sapphire substrate employed in the experiment possesses a distinctive structure characterized by protrusions arranged in a matrix pattern. This unique substrate structure influences the formation of ZnO nanoflowers, resulting in a matrix-like pattern resembling an array of ZnO nanoflowers. Upon examining the growth results presented in Figure 4, it is observed that with increasing growth time, the width of the hexagonal edge lines decreases. When the growth time reaches one hour, ZnO nanoflowers grown from different protrusions begin to overlap, leading to the merging of adjacent hexagonal shapes. Consequently, the edge lines between the hexagonal shapes become indistinguishable or invisible due to the overlapping growth. As a result, the original structure of ZnO nanoflower matrix (NFM) becomes indiscernible, as illustrated in Figure 4c. To quantify the surface characteristics of ZnO NFM, the specific surface areas were determined using the BET method. The measurements revealed specific surface areas of 11.6, 14.9, and 17.8 m^2^/g for synthesis times of 30, 45, and 60 min, respectively. Based on these findings, we propose a hypothesis that the increase in the specific surface area of ZnO NFM is attributed to the longer ZnO nanorod length achieved through extended synthesis times. The longer nanorods contribute to a higher surface-to-volume ratio, resulting in an increased specific surface area.

**Figure 5:**
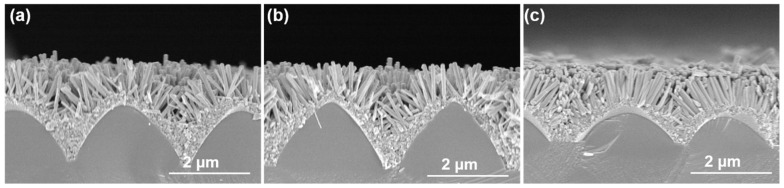
SEM cross-sectional of synthesized ZnO NRs, (**a**) 30, (**b**) 45, and (**c**) 60 min.

Figure 5 displays the cross-sectional view of synthesized ZnO nanoflower-like structures (NFMs) using scanning electron microscopy (SEM). The measurements were conducted for synthesis times of 30, 45, and 60 min, and the obtained results are presented in Figure 5a–c, respectively. For the synthesis time of 30, 45, and 60 min, the average lengths of ZnO NFMs were determined to be 343, 430, and 519 nm, respectively. The measured longest and shortest lengths were found to be 328 and 367 nm, 418 and 451 nm, and 505 and 542 nm, respectively. Additionally, the corresponding average diameters of ZnO NFMs were measured to be 108, 125, and 141 nm, and the largest and smallest diameters recorded were 104 and 113 nm, 120 and 128 nm, and 137 and 146 nm, respectively. Table 2 gives the relevant results and the relationship between density and aspect ratio of ZnO NFMs with growth times of 30, 45, and 60 min. The phenomenon of two adjacent ZnO NRs combining into a single nanorod during the growth process contributes to this relationship. With longer growth times, there is a higher probability of nanorod combination, resulting in increased nanorod diameters but reduced overall density of ZnO NFMs. 

This behavior is consistent with the findings presented in Figure 4 and Figure 5, which show the progressive merging and enlargement of ZnO NRs with longer growth times. Utilizing the parameters mentioned above, the total surface area and volume of ZnO NRs grown on the protrusion sapphire substrate can be estimated, as presented in Table 2. For growth times of 30, 45, and 60 min, ZnO NRs densities were 127, 95, and 52 per μm^2^, respectively. The estimated surface areas of ZnO NRs within an area of 1 μm^2^ were 30.3, 40.59, and 51.65 μm^2,^ for the respective growth times. ZnO NRs grown on the protrusion sapphire substrate exhibit a nanoflower structure, resulting in a significantly larger total surface area than ZnO NRs grown in an upward direction, as illustrated in Figure 4. Consequently, these ZnO NRs possess a larger contact area and can react with gases more effectively, thereby enhancing the sensing characteristics of gas sensors. The top areas of ZnO NRs grown on 1 μm^2^ protrusion sapphire substrates were measured to be 0.76, 1.01, and 1.29 μm^2^, respectively, while their corresponding volumes grown on the same substrates were 3.27, 5.07, and 7.28 μm^3^, respectively.

**Figure 6:**
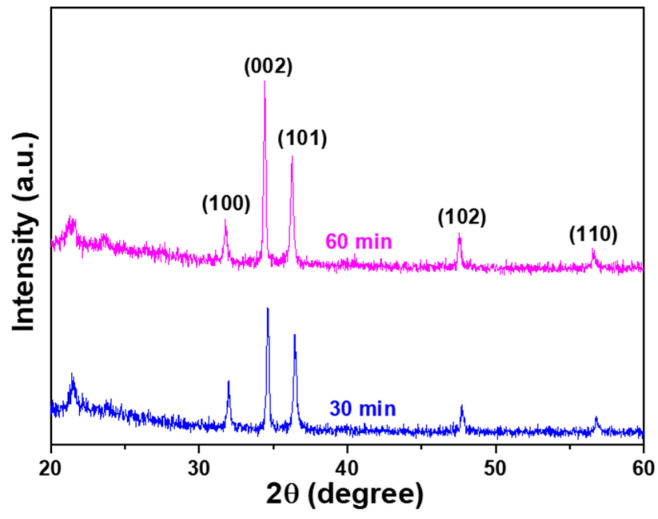
XRD patterns of ZnO NRs synthesized on the sapphire protrusion substrate as the synthesis time of 30 and 60 min.

The X-ray diffraction (XRD) patterns of ZnO NRs synthesized on the sapphire protrusion substrate under different synthesis times were measured, and the results are displayed in Figure 6. Comparing these results with Figure 3b, it can be observed that the main diffraction peak shifted from the (101) plane to the (002) plane. Additionally, diffraction peaks corresponding to the (100), (102), and (110) planes were also observed. Furthermore, Figure 6 illustrates that the diffraction intensity of the (002) plane increased significantly with synthesis time, indicating a preferred orientation along the c-axis of ZnO NRs. The diffraction intensities of the other four planes, namely (100), (101), (102), and (110), also exhibited slight increases with longer synthesis times. These findings suggest that the crystal structure of ZnO NRs is influenced by the growth duration, leading to changes in the dominant diffraction planes and their corresponding intensities. The crystalline properties of ZnO NRs can be determined using the biaxial strain model [18]. In this model, stress is a crucial factor for the stability of ZnO NRs. In the case of ZnO NRs grown on the sapphire protrusion substrate, which exhibit a flower-shaped morphology as depicted in Figure 4 and Figure 5, the stress values are positive, indicating tensile forces acting on the nanorods. From Figure 6, it can be observed that the 2θ value, FWHM value, grain size, and strain of ZnO nanoflower-like structures (NFMs) synthesized for 60 min were measured as 36.21°, 0.035°, 36.9 nm, and 1.502 GPa, respectively. These parameters provide insights into the crystal properties and mechanical stability of 60 min-synthesized ZnO NFMs.

**Figure 7:**
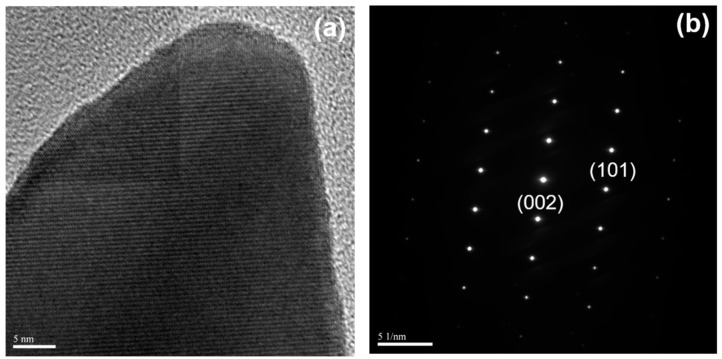
TEM images taken on a single ZnO nanorod synthesized on the sapphire protrusion substrate as the synthesis time of 60 min. (**a**) high-resolution TEM micrograph and (**b**) electron diffraction pattern.

High-resolution electron microscopy was utilized to conduct measurements on the lattice fringe widths. The high-resolution TEM micrographs were captured using a field emission gun operated at an acceleration voltage of 200 kV. The obtained results are presented in Figure 7a, while Figure 7b illustrates the processed fast Fourier transformation of the corresponding image. The observed lattice spacing measures 2.58 Å, indicating the presence of (002) planes within the Wurtzite structure.

**Figure 8:**
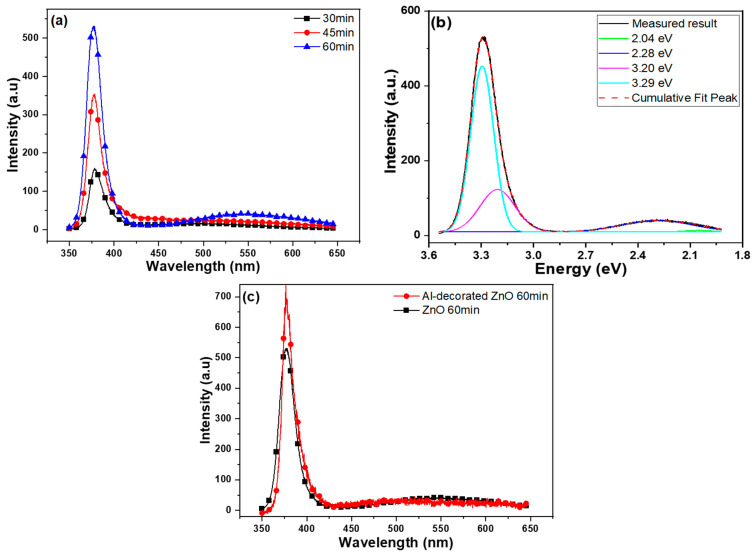
PL spectrum of ZnO NFMs with (**a**) different synthesis times, (**b**) with a synthesis time of 60 min and the fitting results using the sum of four Gaussian functions, and (**c**) comparison of 60-min synthesized ZnO NFMs without and with Al decoration.

The photoluminescence (PL) spectra of ZnO NFMs synthesized at different times are shown in Figure 8a, where a UV light of 325 nm was used as the excitation source. The spectra exhibit two distinct emission peaks. The first peak, denoted as I_UV_, is located at a wavelength of approximately 380 nm and exhibits a strong intensity. This peak arises from the recombination of free excitons [19], indicating a near-band-edge emission characteristic of ultraviolet (UV) light. It is a typical emission observed in ZnO materials. The second peak, spanning from approximately 450 nm to 600 nm, corresponds to a green light emission (I_G_). This emission is attributed to the presence of various defects within synthesized ZnO NFMs. The defects in the material contribute to the energy levels and transitions that result in the emission of green light. Overall, the PL spectra provide valuable information about the optical properties and defect states of ZnO NFMs synthesized at different time durations. As shown in Figure 8a, the I_G_ value, corresponding to the green light emission, increases with the growth time of ZnO NFMs. This indicates that the number of defects in ZnO NFMs increases as the growth time extends. To understand the underlying reasons for this observation, further investigations are needed. Previous studies have indicated that different mechanisms can lead to various emissions in ZnO-based materials [20,21].

In this context, Figure 6 demonstrates that both the I_UV_ and I_G_ values increase with the growth time. The I_UV_ peaks are observed at wavelengths of 378.2, 378.0, and 377.8 nm for growth times of 30, 45, and 60 min, respectively. To understand the specific mechanisms responsible for the emissions, further analysis and characterization are required. These could include studying the defect types and concentrations, investigating the influence of growth conditions, and examining the impact of structural changes on the optical properties of ZnO NFMs. Additionally, the full width at half maximum values for the I_UV_ peaks in the PL spectra were measured. For growth times of 30, 45, and 60 min, the FWHM values were 19.1 nm (371.5–390.6 nm), 29.5 nm (369.3–397.8 nm), and 37.5 nm (367.4–384.9 nm), respectively. These results indicate that as the growth time increases, the FWHM value also increases. It is worth noting that this result differs significantly from the previous observation of the IG values, which showed a decrease with longer growth times. One possible explanation for this discrepancy is the difference in the substrate structure used in this study compared to previous research [22]. The substrate structure can have a significant impact on the growth process and the resulting optical properties of ZnO NFMs, leading to varying trends in the PL spectra.

In Figure 8b, the PL spectrum of ZnO NFMs synthesized at 60 min is plotted with energy (eV) as the x-axis. To analyze this spectrum, a fitting procedure was performed on the merged emission profile in the range of approximately 3.54–1.91 eV (350–650 nm). The fitting was carried out using four Gaussian functions positioned at approximately 3.29, 3.20, 2.28, and 2.04 eV, respectively. It is important to note that the intensity of the band at 2.04 eV, attributed to the recombination of $V_{o}^{++}$ ions with delocalized electrons [23], was found to be small in the fitting result, and its effect could be neglected. Purbayanto et al. discovered that the emission peak at 3.251 eV is associated with two-electron satellite transitions and donor-acceptor pair transitions [24]. The peaks at 3.29 eV, 3.20 eV, and 2.28 eV are believed to be caused by near-band-edge emission, zinc vacancies (V_Zn_), and interstitial oxygen (O_i_), respectively [20,21]. However, in 60 min-synthesized ZnO NFMs, the peaks corresponding to oxygen vacancy (V_O_) with an energy gap (Eg) of approximately 1.62 eV, antisite defects (O_Zn_) with Eg of ~2.38 eV, interstitial zinc (Zn_i_) with Eg of ~2.90 eV, and zinc vacancy (V_Zn_) with Eg of ~3.06 eV were not observed. 

In Figure 8a, it is evident that the intensity of the green emission peak increases with the growth time of ZnO NFMs. This suggests that as the synthesis time increases, the amount of interstitial oxygen in the grown ZnO NFMs decreases. The intensities of I_G_ and I_UV_ and the I_G_/I_UV_ ratio of ZnO NFMs were measured as a function of synthesis time. With the increase in growth time from 30 min to 60 min, the intensity of the IUV value increased from 160 to 529 (a.u.), while the IG value increased from 16.2 to 41.6. Consequently, the I_G_/I_UV_ ratio decreased from 0.101 to 0.079. Figure 8c presents a comparison between 60 min-synthesized ZnO NFMs without and with Al decoration. The I_UV_ value significantly increased to 710 when Al was used to decorate ZnO NFM, while the I_G_ value showed no apparent change. This result from Figure 7c suggests that Al decoration has a noticeable effect on the I_UV_ value of a ZnO NFM. Consequently, the Al decoration is likely to impact the sensing properties of synthesized ZnO NFMs.

**Figure 9:**
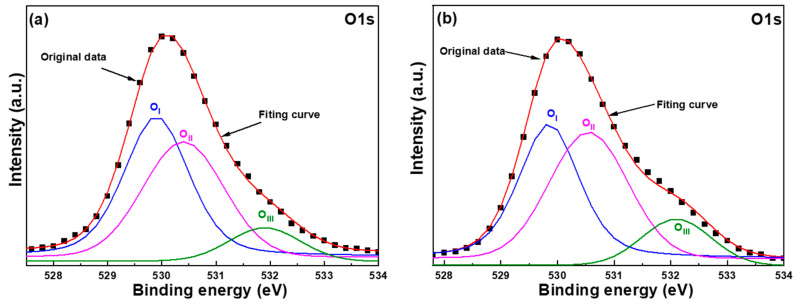
The O_1s_ peaks of ZnO NFMs using different synthesis time, (**a**) 30 min and (**b**) 60 min.

We conducted X-ray photoelectron spectroscopy (XPS) to analyze the chemical bonding state of oxygen in order to investigate the effect of synthesis time. This analysis helps establish a relationship between defects caused by different synthesis times and the optical properties of ZnO NFMs. The O1s peak of ZnO NFMs was measured for samples synthesized for 30 min and 60 min, and the corresponding results are illustrated in the accompanying diagrams. The typical surface O_1s_ peaks of ZnO NFMs are centered at 530.1 ± 0.1 eV and fitted by three Gaussian components of O_I_, O_II_, and O_III_ peaks, as Figure 9a,b shows, which are centered at 529.7 ± 0.1 eV, 530.2 ± 0.2 eV, and 532.2 ± 0.2 eV, respectively. For ZnO NFMs, the bond energy for components of O_I_, O_II_, and O_III_ peaks are caused by the Zn^2+^-O^2−^ bond, the oxygen vacancies, and the chemical absorption of oxygen on the surfaces [25]. Table 3 presents a comparison of the areas of O_1s_ peaks measured from the XPS spectra of ZnO NFMs as a function of synthesis time. With an increase in synthesis time from 30 min to 60 min, the area of the OI peak decreases from 48.89% to 45.62%, the area of the OII peak increases from 41.48% to 43.08%, and the area of the OIII peak increases from 9.63% to 11.30%. The observed increase in the area of the OII peak of ZnO NFMs with the increase in synthesis time provides evidence that the oxygen vacancies in ZnO NFMs increase over time. This suggests that the defects in ZnO NFMs also increase with longer synthesis times. Furthermore, since the oxygen vacancies increase with synthesis time, these results indirectly support the finding that the intensity of the I_G_ value in ZnO NFMs increases with synthesis time, which is consistent with the measured results shown in Figure 8.

**Figure 10:**
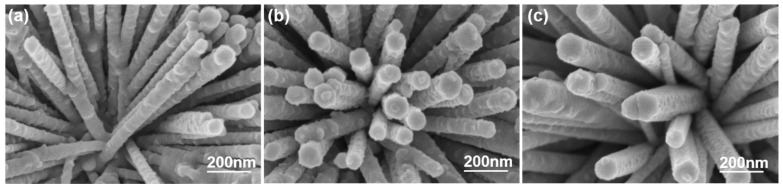
Al-decorated (**a**) 30-, (**b**) 45-, and (**c**) 60-min synthesized ZnO NFMs.

In this study, the thermal evaporation method was employed to deposit a 5 nm thick Al film on ZnO NFMs. Subsequently, the Al film was coated and annealed, and the diameter of the formed Al nanoparticles (NPs) was calculated using XRD diffraction patterns. Figure 10a–c present the surface morphologies of Al-decorated ZnO NFMs synthesized for 30, 45, and 60 min, respectively. The diameters of the nano-Al particles on Al-decorated ZnO NFMs were measured as 29.91, 34.8, and 42.7 nm for the respective growth times of 30, 45, and 60 min, respectively.

**Figure 11:**
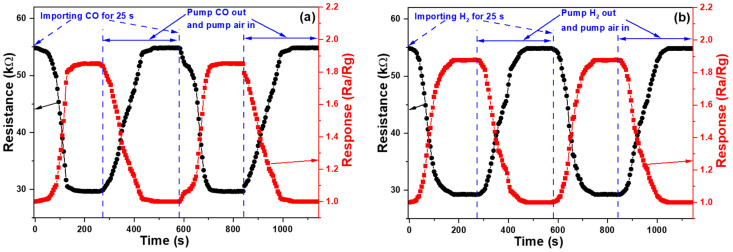
Dynamic analyses of 60 min-growth ZnO NFM gas sensor performed for (**a**) CO and (**b**) H_2_ detections, including changes in resistance values and resistance responses.

When 30 min- and 45-min synthesized ZnO NFMs were used as the sensing devices to detect the CO (H_2_) gas, they took approximately 237 and 196 s (225 and 188 s, resistance falling time) for the resistance to drop from 252 (248) and 143 (141) kΩ to around 187 (184) and 96.7 (95.3) kΩ at the first cycle. We find that the response time is long, and the response rates for CO (H_2_) are 1.348 (1.347) and 1.479 (1.480), respectively. As compared with the sensing results of 60-min synthesized ZnO NFMs, we have identified two significant issues: the response rates of both groups are relatively lower and the resistance values of both groups are relatively high, leading to considerable response rate and resistance drifts during testing processes. Consequently, we have excluded these two sets of sensors from further use and comparison. Figure 11 illustrates the CO and H_2_ gas sensing properties of undecorated ZnO NFM grown on a sapphire convex substrate for 60 min. In Figure 11a, when 2000 ppm of CO was introduced, it reacted with the oxygen ions adsorbed on ZnO NFM, releasing electrons to the zinc oxide and causing the resistance to decrease. It took approximately 130 s for the resistance to drop from 54.83 kΩ to around 30.52 kΩ at the first cycle. Subsequently, after pumping out the CO and introducing air, the resistance value gradually returned to about 51.65 kΩ after approximately 115 s (resistance rising time). These test results were also used to evaluate the reproducibility of the component, which involved testing the component repeatedly for 10 cycles to determine whether the resistance value and response rate remained stable. The average resistance values for the 10 (100) cycles were found to be 54.9 (55.3) and 31.2 (33.4) kΩ for the high and low values and the average resistance falling and rising times were 132 (134) and 118 (121) s, respectively. These results demonstrate that the gas sensors fabricated using ZnO NFM can be used repeatedly. The comparisons of response properties of ZnO NFM-based sensors under different synthesis times at the first measurement round are compared in Table 4.

In Figure 11b, the oxygen ions began to combine with the H_2_ molecules, which were introduced at a concentration of 2000 ppm, adsorbed on ZnO NFM and released electrons to ZnO NFM, and as a result, the resistance started to decrease. As shown Figure 11a, the resistance decreased from 54.86 kΩ to approximately 30.70 kΩ in about 130 s at first cycle. Subsequently, the H_2_ was removed, and air was introduced, which caused the resistance value to recover to approximately 51.58 kΩ after 125 s. These test results were also subjected to device reproducibility testing, which involved repeating the test for the gas sensors ten times to determine whether the device’s resistance values and response change rates were stable. It was found that the average high and low resistance values for the 10 (100) cycles were 55.6 (53.7) and 56.1 (33.1) kΩ and the average resistance falling and rising times were 133 (136) and 128 (131) s, respectively, which demonstrated that the device was also reusable for H_2_ sensing. The response rates of CO and H_2_ sensing for 60 min-growth undecorated ZnO NFM are also shown in Figure 11. The maximum response rates of CO and H_2_ for sensing at first cycle were 1.796 and 1.788, the average maximum response rates for the 10 cycles of CO and H_2_ sensing were 1.759 (which is 95.1% of 1.796) and 1.782 (which is 94.9% of 1.788), and the average maximum response rates for the 100 cycles of CO and H_2_ sensing were 1.656 (89.5%) and 1.695 (90.3%), respectively. 

**Figure 12:**
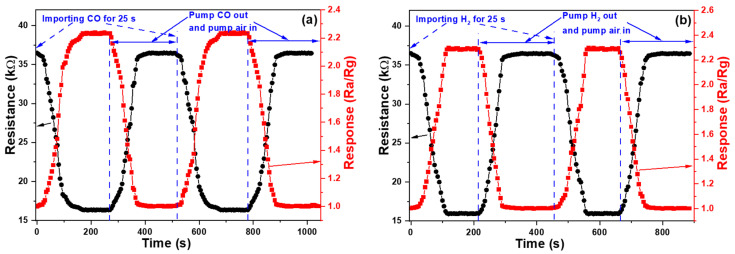
Dynamic analyses of Al-decorated 60 min-growth ZnO NFM gas sensors performed for (**a**) CO and (**b**) H_2_ detections, including changes in resistance values and resistance responses.

Figure 12 shows the sensing characteristics of ZnO NFM gas sensors decorated with Al NPs and grown for 60 min. As Figure 12a shows, after 2000 ppm of CO was introduced, the resistance began to decrease, and it took about 120 s to drop from 34.5 to about 17.2 kΩ. Then the CO was pumped out and air was introduced, and the resistance value returned to about 34.34 kΩ after about 90 s. The average high and low resistance values for the 10 (100) cycles CO sensing were 34.8 (35.2) and 17.9 (18.9) kΩ and the average resistance falling and rising times were 122 (124) and 91 (93) s, respectively, which demonstrated that the device was also reusable for CO sensing. It can also be seen from Figure 12b that after the introduction of 2000 ppm of H_2_, the resistance started to decrease, and it took about 105 s to drop from 36.5 kΩ to about 16.9 kΩ. Then H_2_ was pumped out and air was introduced, and the resistance value returned to about 36.0 kΩ after about 65 s. The average high and low resistance values for the 10 (100) cycles H_2_ sensing were 37.1 (38.0) and 17.8 (18.9) kΩ and the average resistance falling and rising times were 106 (108) and 64 (66) s. Comparing the sensing results in Figure 11 and Figure 12, we find that Al-decorated ZnO NFM gas sensors have lower resistance, faster response times, and higher response rates in the sensing processes of CO and H_2_ gases. The maximum response rates of CO and H_2_ for sensing at first cycle were 2.006 and 2.160, the average maximum response rates for the 10 cycles of CO and H_2_ sensing were 1.942 (which is 96.8% of 2.006) and 2.087 (which is 96.6% of 2.160), and the average maximum response rates for the 100 cycles of CO and H_2_ sensing were 1.862 (92.8%) and 2.009 (93.0%), respectively. These results in Figure 11 and Figure 12 prove that the Al-decorated ZnO NFMs have a higher response rate, a shorter response time, and higher cycling stability. 

The inductive properties of metal oxide semiconductors are mainly due to the phenomenon of charge transfer caused by the adsorption and desorption of oxygen atoms on metal oxides, which in turn changes the electrical properties [3,25]. Oxygen atoms adsorb on the surface of the oxide structure and trap electrons, forming oxygen ions that can reduce the electrical conductivity of metal oxide semiconductors. When the metal oxide semiconductors are exposed to the environment of reducing gases such as CO, the CO gas molecules will react with the adsorbed oxygen ions to form carbon dioxide CO_2_, which will lead to the release of electrons and improve the conductivity. Takata et al. found that oxygen ions exist in the form of O^2−^ in low temperature environments (below 100 °C) [26]. When the temperature is between 100–300 °C, it will exist in the form of O^−^ ions, and when the temperature is higher than 300 °C, it will transform into O^2−^ ions [27]. The reaction of CO to oxygen ions at various temperatures is shown in the following reactions.

Below 100 °C: 2CO + O_2_^−^ → 2CO_2_ + e^−^ (3)

Between 100–300 °C: CO + O^−^ → CO_2_ + e^−^ (4)

Above 300 °C: CO + O^2−^ → CO_2_ + e^−^ (5)

Because the test temperature is 200 °C, the main reason for the drop in resistance of ZnO NFM gas sensors is the reaction Formula (4). The induction mechanism of H_2_ is the same, mainly that the electrons reduced on the surfaces of ZnO NRs will be released to the conduction band and help to increase the current flowing through ZnO NRs.

A larger specific surface area provides more sites for gaseous molecules to be adsorbed, while a smaller crystal size (approximately equal to the Debye length λ_D_) results in total depletion, leading to higher sensitivity. The Debye length λ_D_, which is calculated using the equation λ_D_ = {2k_B_T/e^2^N_d_}^1/2^, is strongly influenced by the carrier density (N_d_). Here, k_B_ is the Boltzmann constant, T is the absolute temperature, and e is the electron charge [5]. Therefore, by controlling the carrier density, the effective Debye length of the oxide can be adjusted to maximize the gas sensing response. For instance, increasing the carrier density can decrease the Debye length, which is equivalent to increasing the effective crystal size of the material. As noted earlier, the Debye length depends on the concentration of carriers, which means that the introduction of various metal decorations on the surfaces of the fabricated samples will change the concentration of carriers. However, regardless of the type of metal used, decorating the surface of ZnO NRs with metal will elevate the carrier concentration (electrons in the metal), and that will lead to a shorter Debye length and improve efficiency in gas sensing.

## 4. Conclusions

The 2θ value, FWHM value, grain size, and strain of 60 min-synthesized ZnO NFMs were 36.21°, 0.035°, 36.9 nm, and 1.502 Gpa, respectively. The PL spectrum of 60 min-growth ZnO NFMs was fitted to a sum of four Gaussian functions located at approximately 3.29, 3.20, 2.28, and 2.04 eV, which were caused by the near-band-edge emission, the zinc vacancies, the interstitial oxygen, and the recombination of $V_{o}^{++}$ ions with the delocalized electrons, respectively. When undecorated ZnO NFM was used to sense CO (H_2_) gas, the average resistance values were found to be 52.3 (52.1) and 31.4 (31.5) kΩ for the high and low values and the average resistance falling and rising times were 132 (133) and 118 (128) s, and the average maximum response rates of CO and H_2_ sensing were 1.842 and 1.873, respectively. When Al-decorated ZnO NFMs were used to sense CO (H_2_) gas, the average resistance values were found to be 34.9 (34.6) and 17.5 (17.3) kΩ for the high and low values, and the average resistance falling and rising times were 122 (106) and 91 (64) s, respectively. The average maximum response rates of undecorated ZnO NFM for CO and H_2_ sensing were 1.842 and 1.873, and the average maximum response rates of al-decorated ZnO NFM for CO and H_2_ sensing were 2.228 and 2.315, respectively. This study proves that Al-decorated ZnO NFM gas sensors have the better sensing characteristics than undecorated ZnO NFM gas sensors, because they have the faster response times and higher response rates during the CO and H_2_ sensing processes.

## Figures and Tables

**Table 1:** Concentration parameters of the measured gas.

Preset Concentration	Inlet Gas Flow	Inlet Gas Time
100 ppm	50 sccm	5 s
500 ppm	200 sccm	6.25 s
1000 ppm	200 sccm	12.5 s
1500 ppm	200 sccm	18.75 s
2000 ppm	200 sccm	25 s

**Table 2:** Morphological properties of synthesized ZnO NFMs as a function of synthesis time.

Synthesis Time	30 min	45 min	60 min
Diameter (D, nm)	108	125	141
Length (L, nm)	343	430	519
L/D ratio	3.2	3.4	3.7
Density (μm^−2^)	127	95	52
Total surface area (μm^2^)	30.3	40.59	51.65
Total top surface area (μm^2^)	0.76	1.01	1.29
Total volume (μm^3^)	3.27	5.07	7.28

**Table 3:** Areas of O_1s_ peaks of ZnO NFMs as a function of synthesis time.

Synthesis Time	30	45	60
O_I_	48.89%	47.29%	45.62%
O_II_	41.48%	42.42%	43.08%
O_III_	9.63%	10.29%	11.30%

**Table 4:** Comparisons of response properties of ZnO NFM-based sensors under different synthesis times at the first test.

	CO			H_2_		
synthesis time	30	45	60	30	45	60
falling time	237 s	196 s	130 s	225 s	188 s	130 s
high resistance	252 kΩ	143 kΩ	54.83 kΩ	248 kΩ	141 kΩ	54.86 kΩ
low resistance	187 kΩ	96.7 kΩ	30.52 kΩ	184 kΩ	95.3 kΩ	30.70 kΩ
response rate	1.348	1.479	1.796	1.347	1.479	1.787

## Data Availability

Not applicable.
